# Supplementary material for: Automated measurement of anteroposterior diameter and foraminal widths in MRI images for lumbar spinal stenosis diagnosis
Source: PLoS One. 2020 Nov 2;15(11):e0241309. doi: 10.1371/journal.pone.0241309 (PMC7605707; doi:10.1371/journal.pone.0241309)
Supplement: S1 File — (DOCX) [file pone.0241309.s001.docx]

**Algorithm 1.** EvolveBoundaries

Inputs: Boundary Grid $B^{'}$

Search width $w$

Union of all boundary points sets$S$

Output: Evolved Boundary Grid ${B^{'}}_{out}$

**let** $w_{s}=2^{w}$

**let** $h=-w_{s}:2: w_{s}$ // h = [-ws, –ws+2, –ws+4, … 0, 2, 4, … ws]

**let** $h_{1}=-w_{s}:2: 0$ // h1 = [-ws, –ws+2, –ws+4, … 0]

**let** $h_{2}=0:2: w_{s}$ // h2 = [0, 2, 4, … ws]

**let** $i_{c}=w+1;$ // w+1 is the center of the index vector marking current pixel

// hence the index vector is [1, 2, … w+1, … 2w+1]

**for** $\forall(r,c)$ where $(r,c)\in S$

**let** $a = \mathrm{sign} (B^{'}\left( r,c \right))$;

**if** $a==0$ **then** $a=1$

**if** ($r$ **mod** 2) == 0 // row is an even number hence vertical edge

**let** $g=B^{'}\left( r+h,c \right)\times a;$ // g is a vector with the same length as h

**let** $i=\arg\max g$ // returns the index of max element

**let** $d=i_{c}-i$ // distance to the highest gradient

**if** $d>0$ // max gradient is above the current pixel

**let** $d_{v}=1:2:2d$ // all cells from current to max position

$B^{'}\left( r-d_{v},c \right)=B^{'}\left( r+1,c \right);$

**else if** $d<0$ // max gradient is below the current pixel

$B^{'}\left( r+d_{v},c \right)=B^{'}\left( r-1,c \right);$

**else if** ($c$ **mod** 2) == 0

// col is an even number hence horizontal edge

**let** $g=B^{'}\left( r,c+h \right)\times a;$ // g is a vector with the same length as h

**let** $i=\arg\max g$ // returns the index of max element

**let** $d=i_{c}-i$ // distance to the highest gradient

**if** $d>0$ // max gradient is above the current pixel

**let** $d_{v}=1:2:2d$ // all cells from current to max position

$B^{'}\left( r,c-d_{v} \right)=B^{'}\left( r,c+1 \right);$

**else if** $d<0$ // max gradient is below the current pixel

$B^{'}\left( r,c+d_{v} \right)=B^{'}\left( r,c-1 \right);$

${B^{'}}_{out}=B^{'};$

**Algorithm 2.** SubPixelBoundaryEvolution

Inputs: Input Image $I$

Width of Gaussian smoothing kernel $\sigma$

Initial Label Image ${}_{0}$

Search width $w$

Iteration averaging window $n$

Standard Deviation threshold $\tau$

Number of up samplings $k$

Output: Evolved Label Image

**let** $={}_{0}$

**construct** $G_{\sigma}$ // a Gaussian kernel with width $\sigma$

**let** $D$ = Ø // an empty sequence to store difference values,

**for** $i=1:k$

**let** $g_{h}=\nabla_{h}(G_{\sigma}*I)$ // Horizontal gradient image

**let** $g_{v}=\nabla_{v}(G_{\sigma}*I)$ // Vertical gradient image

**construct** $B'$ from ,$g_{h}$ and $g_{v}$ // use Eq.1

**construct** $B_{S}$ from $B'$

**select** $S$ from $B'$ // S is a set of important boundary points to evolve

**for** $t$=1:$t_{max}$ // we put a hard cap on the number of iterations

${B^{'}}_{out}$ = EvolveBoundaries($B^{'},w,S$);

**let** $\partial=\left| {B^{'}}_{out}\neq B^{'} \right|$ // number of pixel changes between ${B^{'}}_{out}$ and $B^{'}$

**append** $\partial$ to $D$ // appending $\partial$ to the end of $D$ sequence

**if** $\mathrm{card} (D)\geq$ $n$ // length of D exceeds n-1

**let** $v=\sqrt{\frac{\sum\left| D-\bar{D} \right|}{n}}$ // use standard deviation as a measure of convergence

**if** $v<\tau$

**break**; // converges, so break from the loop

**else**

$D\left[ 1 \right]=\emptyset$ // remove the oldest value in $D$ and ensuring $\mathrm{card} (D)$ < $n$

= downsample($B^{'},2$) // we retrieve the label image from $B^{'}$ simply by // downsampling it at its every odd pixel location

**if** $i\neq k$

$I$ = upsample($I,2$) // up sample the input image using cubic interpolation

= upsample($,2$) // up sample the label image using nearest interpolation
