# Supplementary material for: Automated measurement of anteroposterior diameter and foraminal widths in MRI images for lumbar spinal stenosis diagnosis
Source: PLoS One. 2020 Nov 2;15(11):e0241309. doi: 10.1371/journal.pone.0241309 (PMC7605707; doi:10.1371/journal.pone.0241309)
Supplement: S2 File — (DOCX) [file pone.0241309.s002.docx]

**The dataset and corresponding MATLAB code to reproduce the results can be found on Mendeley Data:**

1. Sudirman, Sud; Natalia, Friska (2020), “MATLAB source code for automated measurement of anteroposterior diameter and foraminal widths in MRI images for lumbar spinal stenosis diagnosis”, Mendeley Data,

published version, <http://dx.doi.org/10.17632/zwd3hgr6gg.2>

preview version, <https://data.mendeley.com/datasets/zwd3hgr6gg/draft?a=70e4beb9-4e64-4fe9-9104-ddeace640fca>

1. Sudirman, Sud; Al Kafri, Ala; Natalia, Friska; Meidia, Hira; Afriliana, Nunik; Al-Rashdan, Wasfi; Bashtawi, Mohammad; Al-Jumaily, Mohammed (2019), “Lumbar Spine MRI Dataset”, Mendeley Data, v2, <http://dx.doi.org/10.17632/k57fr854j2.2>
2. Sudirman, Sud; Al Kafri, Ala; Natalia, Friska; Meidia, Hira; Afriliana, Nunik; Al-Rashdan, Wasfi; Bashtawi, Mohammad; Al-Jumaily, Mohammed (2019), “Label Image Ground Truth Data for Lumbar Spine MRI Dataset”, Mendeley Data, v2, <http://dx.doi.org/10.17632/zbf6b4pttk.2>
3. Sudirman, Sud; Al Kafri, Ala; Natalia, Friska; Meidia, Hira; Afriliana, Nunik (2019), “MATLAB source code for developing Ground Truth Dataset, Semantic Segmentation, and Evaluation for the Lumbar Spine MRI Dataset”, Mendeley Data, v2, <http://dx.doi.org/10.17632/8cp2cp7km8.2>
4. Sudirman, Sud; Al Kafri, Ala; Natalia, Friska; Meidia, Hira; Afriliana, Nunik; Al-Rashdan, Wasfi; Bashtawi, Mohammad; Al-Jumaily, Mohammed (2019), “Radiologists Notes for Lumbar Spine MRI Dataset”, Mendeley Data, v2, <http://dx.doi.org/10.17632/s6bgczr8s2.2>

By following the instructions set out in the first (or second) URL and using only the data that are available at the above URLs, other researchers should be able to replicate the results in our study.
