# Supplementary material for: Automated measurement of anteroposterior diameter and foraminal widths in MRI images for lumbar spinal stenosis diagnosis
Source: PLoS One. 2020 Nov 2;15(11):e0241309. doi: 10.1371/journal.pone.0241309 (PMC7605707; doi:10.1371/journal.pone.0241309)
Supplement: Attachment — Submitted filename: Response to Reviewers.docx [file pone.0241309.s004.docx]

**Response to the Reviewers**

First of all, we would like to thank the reviewers for reviewing the latest version of our paper. Your comments and feedback are very valuable for improving the quality of the paper. We have made the minor but necessary change to our paper based on your feedback. Please find below our response to specific areas of the paper and the outline of the changes that we made. I hope that the change and our explanation are to your satisfaction.

**Reviewer #1.**

1. If the authors have adequately addressed your comments raised in a previous round of review and you feel that this manuscript is now acceptable for publication, you may indicate that here to bypass the “Comments to the Author” section, enter your conflict of interest statement in the “Confidential to Editor” section, and submit your "Accept" recommendation.

Reviewer #1: (No Response)

________________________________________

2. Is the manuscript technically sound, and do the data support the conclusions?

The manuscript must describe a technically sound piece of scientific research with data that supports the conclusions. Experiments must have been conducted rigorously, with appropriate controls, replication, and sample sizes. The conclusions must be drawn appropriately based on the data presented.

Reviewer #1: (No Response)

_______________________________________

3. Has the statistical analysis been performed appropriately and rigorously?

Reviewer #1: (No Response)

________________________________________

4. Have the authors made all data underlying the findings in their manuscript fully available?

The PLOS Data policy requires authors to make all data underlying the findings described in their manuscript fully available without restriction, with rare exception (please refer to the Data Availability Statement in the manuscript PDF file). The data should be provided as part of the manuscript or its supporting information, or deposited to a public repository. For example, in addition to summary statistics, the data points behind means, medians and variance measures should be available. If there are restrictions on publicly sharing data—e.g. participant privacy or use of data from a third party—those must be specified.

Reviewer #1: (No Response)

________________________________________

5. Is the manuscript presented in an intelligible fashion and written in standard English?

PLOS ONE does not copyedit accepted manuscripts, so the language in submitted articles must be clear, correct, and unambiguous. Any typographical or grammatical errors should be corrected at revision, so please note any specific errors here.

Reviewer #1: (No Response)

________________________________________

6. Review Comments to the Author

Please use the space provided to explain your answers to the questions above. You may also include additional comments for the author, including concerns about dual publication, research ethics, or publication ethics. (Please upload your review as an attachment if it exceeds 20,000 characters)

Reviewer #1: The authors has done extensive revision to address the reviewers comments. I have only a minor suggestion to shorten the conclusion and convey the main contribution/message/conclusion of this paper.

**Our response:**

Thank you for confirming that our last revision has addressed all the reviewers’ comments. We have made the suggested minor change to the conclusion by making it more concise to emphasise the main message of the paper. Specifically, we removed the inclusion of specific results that were already provided in the abstract.

The last four sentences in the conclusion that state:

*Our experiments show that our method can locate important points that are on region boundaries between 0.37 – 1.36 mm of the expert-located reference points. The average error of the calculated right and left foraminal distances relative to their expert-measured distances are 0.28 mm (p = 0.92) and 0.29 mm (p = 0.97), respectively. The average error of the calculated AP diameter relative to their expert-measured diameter is 0.90 mm (p = 0.92). The method also achieves 96.7% agreement with an expert opinion on determining the severity of the IVD herniation.*

have been replaced with:

*Our experiments show that our method can locate all the important points that are on region boundaries accurately. This allows accurate calculation of the AP diameter, which in turn, produces high agreement with the expert’s opinion when determining the severity of the IVD herniations.*

**Reviewer #3.**

1. If the authors have adequately addressed your comments raised in a previous round of review and you feel that this manuscript is now acceptable for publication, you may indicate that here to bypass the “Comments to the Author” section, enter your conflict of interest statement in the “Confidential to Editor” section, and submit your "Accept" recommendation.

Reviewer #3: (No Response)

________________________________________

2. Is the manuscript technically sound, and do the data support the conclusions?

The manuscript must describe a technically sound piece of scientific research with data that supports the conclusions. Experiments must have been conducted rigorously, with appropriate controls, replication, and sample sizes. The conclusions must be drawn appropriately based on the data presented.

Reviewer #3: Partly

_______________________________________

3. Has the statistical analysis been performed appropriately and rigorously?

Reviewer #3: Yes

________________________________________

4. Have the authors made all data underlying the findings in their manuscript fully available?

The PLOS Data policy requires authors to make all data underlying the findings described in their manuscript fully available without restriction, with rare exception (please refer to the Data Availability Statement in the manuscript PDF file). The data should be provided as part of the manuscript or its supporting information, or deposited to a public repository. For example, in addition to summary statistics, the data points behind means, medians and variance measures should be available. If there are restrictions on publicly sharing data—e.g. participant privacy or use of data from a third party—those must be specified.

Reviewer #3: Yes

________________________________________

5. Is the manuscript presented in an intelligible fashion and written in standard English?

PLOS ONE does not copyedit accepted manuscripts, so the language in submitted articles must be clear, correct, and unambiguous. Any typographical or grammatical errors should be corrected at revision, so please note any specific errors here.

Reviewer #3: Yes

________________________________________

6. Review Comments to the Author

Please use the space provided to explain your answers to the questions above. You may also include additional comments for the author, including concerns about dual publication, research ethics, or publication ethics. (Please upload your review as an attachment if it exceeds 20,000 characters)

Reviewer #3: The objective of this paper is to achieve automated diagnosis of lumbar spinal stenosis by measuring the anteroposterior diameter and foraminal widths of lumbar spine. The proposed framework is not novel because it only adopts SegNet and the contour evolution algorithm is a project solution rather than focusing on research innovation.

**Our response:**

We appreciate your comment regarding the novelty of our approach. Although application of SegNet and Contour Evolution algorithms themselves are not novel, we believe we have shown through our extensive set of experiments that our contour evolution technique can provide significant improvement relative to using SegNet alone or the benchmark contour evolution techniques. We believe that our approach could benefit other researchers when they are faced with similar problems. With that I hope our paper can be deemed to have sufficient merit and appropriate for publication.
